# Diet Traps during Eating Disorders among Dentate Patients at an Oral Health Glance

**DOI:** 10.3390/nu15204414

**Published:** 2023-10-18

**Authors:** Elzbieta Paszynska, Amadeusz Hernik, Hélène Rangé, Bennett T. Amaechi, Georgiana S. Gross, Malgorzata Pawinska

**Affiliations:** 1 Department of Integrated Dentistry, Poznan University of Medical Sciences (PUMS), 60-812 Poznan, Poland; 2 Department of Periodontology, Faculty of Odontology, University of Rennes, CHU de Rennes, F-35000 Rennes, France; 3 Institut NUMECAN (Nutrition Metabolism and Cancer), INSERM, INRAE, University of Rennes, F-35000 Rennes, France; 4 Department of Comprehensive Dentistry, School of Dentistry, University of Texas Health San Antonio, San Antonio, TX 78229, USA; 5 Department of Integrated Dentistry, Medical University in Bialystok, 15-276 Bialystok, Poland

**Keywords:** eating disorders, nutrition, oral health, oral hygiene, risk factors

## Abstract

Persons suffering from eating disorders (ED) may often experience a recurrence/persistence symptoms despite the completion of psychiatric therapy. In most cases, their general health status is linked to current nutritional behaviors. Medical professionals, general practitioners (GPs), dieticians, and dentists may see those patients in their practices. At the same time, due to low sense of illness, some patients may delay or never seek professional medical care. The aim of this article is to analyze the main ED types according to dietary behaviors causing oral health problems and discuss oral health complications in affected dentate patients. The second objective is to update oral preventive measures and technological innovations together with active agents for oral hygiene care that might effectively support oral health maintenance during the presence of long-term symptoms. The research method involved a review of clinical reports as a synthesis of the electronic research in the Pubmed, Web of Science, and Google Scholar databases. Based on the research, ED patients were found to present related incidences of oral complications. Studies have reported that the possible course of an ED and comorbidities may be an imbalance in the oral environment. The results showed an association between biological (malnutrition, etc.), behavioral (binge eating episodes, vomiting, acidic diet, poor oral hygiene), and pharmacotherapeutic (addiction, hyposalivation) factors that may threaten oral health. Early diagnosis of the past and present symptoms is essential to eliminate and take control of destructive behaviors. Oral changes need to be tackled with medical insight, and additionally, the perception of dietary interactions is recommended.

## 1. Introduction

Medical professionals, including general practitioners (GPs), dieticians, and dentists, may see eating disorder (ED) patients in their practices. Patients attending medical or dental appointments usually show up for a routine check-up, and EDs can be diagnosed by accident. Those affected by eating disorders are young and often unaware of the seriousness of the complications that can occur as a result of a lack of diagnosis, untreated disease, and dietary errors. Romanos (2012) points out that oral symptoms can appear asymptomatically [1]. Implementing treatment standards, and providing professional medical and psychological help, can happen faster if there is knowledge on and awareness of dietary traps. This increases the chance of not only preventing dangerous health complications manifested in the oral cavity but also improving the prognosis of disease treatment.

The aim of this article is to analyze the main ED types according to the dietary choices that cause oral health symptoms and to discuss the side effects associated with EDs in affected dentate patients. The second goal is to update available oral preventive measures and technological innovations together with active oral hygiene measures and care that might effectively maintain oral health during the presence of long-term ED symptoms. The research method involved a review of the clinical reports on EDs as a synthesis of the electronic research in the Pubmed, Web of Science, Google Scholar databases, addressing patients residing in industrialized countries.

## 2. Eating Disorder Characteristics

### 2.1. Types of Eating Disorders

**Figure 1:**
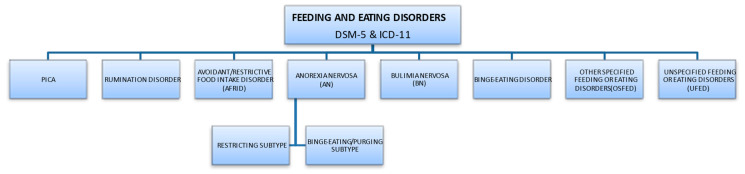
Division of EDs according to DSM-5 and ICD-11 subtypes.

**Figure 2:**
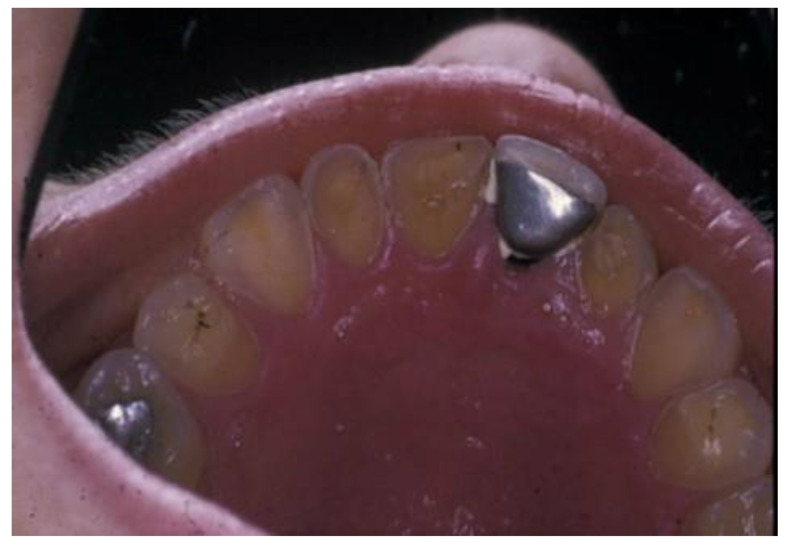
Palatal surfaces of maxillary teeth affected by erosive tooth wear (Courtsey Amaechi BT, UTHSA, San Antonio, TX, USA).

The most important classifications that describe diagnostic criteria are the American Psychiatric Association (APA) guidelines, provided in the Diagnostic and Statistical Manual of Mental Disorders (DSM 5th edition, updated in 2013), and the International Classification of Diseases (ICD-11), approved by the World Health Organization (updated 2019) [2,3]. According to ICD-11, this group of diseases falls under behavioral syndromes associated with physiological disorders and physical factors. According to the APA, eating disorders are characterized by ongoing abnormal behaviors related to feeding and eating, followed by changes in food intake or impaired food absorption, and this in turn causes disorders of physical health or social functioning. The symptoms of the disorder allow a clear diagnosis since the diagnosis of a particular disease entity excludes another. Division of EDs according to DSM-5 and ICD-11 subtypes is presented in Figure 1, [2,4].

### 2.2. ED Risk Factors

Eating disorders are characterized by multiple risk factors, which can be biological, psychological, social, and cultural determinants. Individual theoretical models were considered, referring to socio-cultural determinants (ideal body model, gender role), aspects of personal vulnerability (character and behavioral features, including genetic), family and interpersonal background (disorders occurring in the family, e.g., lack of hierarchy, unclear roles, existence of strong ties, turbulent way of solving problems, perfectionism or overprotection of the family, influences of the environment from the same age group), and influence of traumatic life events (e.g., physical, sexual violence) [5,6]. Risk factors predisposing to the disorder in childhood trigger the first onset of the disease in adolescence [7].

Eating disorders often begin with the selective eating of certain foods or following of specific diets, such as vegetarian diets, that seem easy to accept socially [8]. The image of idealized figures created by social media can also lead to behaviors that promote eating disorders [9]. A contributing factor is perfectionism, an ideal that is an important reference point for the individual [10,11,12]. The following risk groups can be recognized: athletes— including more often female athletes, such as those in cycling, judo, gymnastics, and athletics—and people working in modeling, dance training, the military, catering, and show business [13,14]. Biological backgrounds, e.g., disorders of the serotonergic system, are considered in genetic analysis or as correlations with metabolic and immunological features (including glycemic, lipid) and analysis of the fetal period [7]. Meta-analyses indicate the potential importance of single-nucleotide polymorphisms (SNPs) including serotonin receptor gene and serotonin transporter gene (5-HTR2A, 5-HTT), catechol-O-methyltransferase (COMT) polymorphism (Val158Met), and brain-derived neurotrophic factor (BDNF) polymorphism (Val66Met) [15,16]. The heritability in families with mono-dizygotic twins is estimated to be between 48–74% [17]. Genome-wide association studies (GWASs) have identified a chromosome 12 (12q13.2) locus that is relevant to the inheritance of the disease [18].

Researchers’ attention is drawn to changes in both the hunger and satiety centers as well as to the associated regulation of appetite and metabolism. This includes anorexigenic (appetite-reducing) and orexigenic (appetite-stimulating) substances secreted centrally in the central nervous system and peripherally (by adiponectin and enterohormones). Substances that regulate appetite in the short term (e.g., cholecystokinin and other proteins) and long term (hormones leptin, insulin) and enterohormones that interact with the brain–gut axis (e.g., ghrelin, obestatin, neuropeptide B, vaspin phoenixin, spexin, kisspeptin) are involved in the processes of regulating appetite and metabolism [19,20,21,22].

### 2.3. Epidemiology

Current data show that about 2.9 million people worldwide suffer from anorexia nervosa (AN), with the prevalence in European countries being at the levels of 1–4% (women) and 0.3–0.7% (men) [23,24]. Regarding bulimia nervosa (BN), up to 3% of females and more than 1% of males suffer from this disorder during their lifetime [25]. According to current epidemiological data, EDs are characterized by a long-term course and a high mortality rate [6]. Thus, EDs are an important issue for modern medicine, especially since the incidence rate in Western-civilization countries has been almost declining since the 1970s and the full-blown course is being observed in patients at an increasingly younger age [6,25,26,27]. 

## 3. Common Dietary Intakes and Behavioral Habits Related to Oral Health in ED Patients 

Persons with current or ongoing symptoms of EDs rarely seek out a dietitian for help with their erratic eating habits. These people often visit a medical doctor or dentist to help alleviate a health complication brought on by their dysfunctional eating habits. This section will discuss the food types and practices of patients with ongoing eating disorders. Several types of eating disorders have been described and diagnosed.

Anorexia nervosa: Persons with anorexia nervosa exhibit dietary behaviors that are prompted by their fear of weight gain and distorted body image. Their dietary intakes are consumed in very small portions throughout the day. The food intake is followed by extreme exercise to “burn off” the calories consumed. This form of AN is called the “Restricted Type”. A subset of these patients will binge but purge after a small meal intake to further reduce caloric intake. Most of them purge by inducing vomiting, while others use laxatives. Foods commonly used by these patients are lower-calorie foods generally lower in sugar than the food seen in other types of eating disorders [28,29,30].

Bulimia Nervosa: This form of an ED is presented by eating not only very low-calorie foods. Often, high-calorie food is consumed and followed by purging behavior, particularly vomiting. These foods are consumed rapidly, usually in less than 15–20 min before abdominal discomfort sets in. In other cases, laxatives, fasting, and intense exercise are used as well. The vomiting group had a significantly higher intake of diet cola drinks (low-calorie carbonated drinks) in studies [28,29,30].

Binge Eating Disorder: This eating behavior is most destructive to the oral cavity and upper gastrointestinal tract. Individuals involved select high-calorie foods and follow with purging behavior. If purging is not activated, these people may use laxatives or diuretics and endure extreme exercise activity (Table 1). 

Further proof of deficiencies in dietary intakes by persons with ongoing eating disorders of any type was described in the following studies. A case-controlled study by Johansson et al. [28] listed the types of foods consumed during the active disease. Patients filled out a ‘food recall’ of their dietary intakes. The findings concluded that their diet was significantly higher in caffeine-containing cola soft drinks (carbonated drinks). Also, they noted that sugar-containing foods were reduced, along with a reduction in the lunch meal quantity. The NHANES study reviewed the nutritional adequacy of dietary intake in women with anorexia nervosa. In women from 19 to 30 years of age, a 24 h dietary recall was analyzed and compared to the Dietary Reference Intakes (DRIs). It was concluded that all women were deficient in energy, macronutrients, and micronutrients [29,30].

In conclusion, patients with EDs are at risk for malnutrition, which could have significant general and oral health consequences. It is recommended that these patients be assessed both psychologically and physically by health professionals. Counseling by Registered Dietitians is highly recommended to support improved dietary intakes.

## 4. Oral Complications of ED

### 4.1. Oral Hygiene

Until recently, studies have reported conflicting results regarding oral hygiene and periodontal health conditions in ED patients. On the one hand, the ED sufferer exhibits personality traits supposed to lead to overzealous toothbrushing. On the other hand, they suffer from depressive comorbidity with low interest in oral hygiene practices and a higher risk of periodontal diseases. Indeed, periodontal diseases include reversible and irreversible clinical forms of periodontal tissue destruction, known as plaque-induced gingivitis and periodontitis, respectively. The evidence-based pathogenesis of periodontal diseases emphasizes the role of malnutrition [32,33], substance abuse in particular tobacco smoking and alcohol consumption [34], and anxiety [35]. Unbalanced diets with a high consumption of carbohydrates [36,37] and deficiency in vitamins and minerals are frequent in EDs [38]. In addition, mood and anxiety disorders are commonly associated with EDs [39,40]. Hence, ED patients exhibit dietary habits and comorbidities at high risk of periodontal disease. According to the joint classification of the American Academy of Periodontology and the European Federation of Periodontology, plaque-induced gingivitis is an inflammatory response of the gingival tissues resulting from bacterial plaque accumulation located at and below the gingival margin [41]. Managing gingivitis is a primary preventive strategy for periodontitis. Periodontitis is a chronic multifactorial inflammatory disease associated with dysbiotic plaque biofilms and characterized by the progressive destruction of the tooth-supporting apparatus [42]. Its primary features include the loss of periodontal tissue support, manifested through clinical attachment loss (CAL) and radiographically assessed alveolar bone loss; the presence of periodontal pocketing; and gingival bleeding. Without treatment, periodontitis leads to tooth loss.

Case-control studies comparing oral health conditions in EDs with controls have evaluated plaque control with conflicting results. Two studies in the 1990s using the Plaque Index (PI) from Silness and Loe [43], which is a partial recording system prone to bias, found no difference in plaque accumulation between ED and non-ED subjects [44,45]. The oral hygiene level was better in ED outpatients than in non-ED controls in two studies again using non-full-mouth recorded data [28,46]. Poorer plaque control in ED inpatients compared to non-ED controls was shown in two studies [47,48]. In a study with a subgroup analysis, according to the ED diagnosis, plaque control was worse in patients with AN compared to patients with BN and controls with a Plaque Control Record (PCR), measured at 79%, 64%, and 53%, *p* < 0.01, respectively [47]. Hyposalivation induced by the disease and the psychotropic medications [49] may make plaque control difficult and the lack of salivary mechanical and biological antimicrobial actions can favor plaque accumulation [50,51]. However, in studies, the toothbrushing frequency in people with EDs was not different [52] or even higher than in non-ED controls [47,53].

### 4.2. Periodontal Health

Several observational studies have assessed the periodontal status of ED patients, mostly those suffering from AN and BN. Gingivitis case definitions used were often heterogeneous among studies based on partial-mouth examination approaches with the Gingival Index (GI) [54], leading to no difference [45] and less gingival inflammation [28,46] in ED patients. However, the two studies with full-mouth bleeding scores showed a higher occurrence of plaque-induced gingivitis in ED patients [47,48]. In addition, two well-designed case-control studies found that ED patients present significantly fewer healthy (no gingival bleeding after probing) sextants, measured by the Community Periodontal Index and Treatment Needs (CPITN) [55], compared with non-ED controls, but found no difference in the number of sextants with periodontitis (probing pocket depth > 3mm) [48,56]. These results are in accordance with those of Pallier and collaborators, who observed a higher CAL, with significantly more sites exhibiting gingival recessions in the ED group in comparison with the control group, but no difference in the number of sites with probing pocket depth [47]. To sum up, ED sufferers are not at risk of periodontitis but are at high risk of plaque-induced gingivitis and gingival recessions.

Gingival recession is defined as the apical shift of the gingival margin with respect to the cementoenamel junction; it is associated with attachment loss and with the exposure of the root surface to the oral environment [57]. People with AN and BN have an increased prevalence of gingival recession compared to subjects without EDs of the same age [47,48]. Gingival recessions have multiple maxillary and mandibular localizations. A whitish appearance of the free gingival margin of the recession is frequent as a sign of chemically induced tissue damage by intrinsic and extrinsic acidity. Atypical localizations such as the palatine surfaces of the upper molars are characteristic of ED patients [58]. Toothbrushing frequency is one of the main risk factors for gingival recession along with improper toothbrushing duration and force [57]. This could explain the more frequent generalized gingival recessions observed in ED patients. 

Induced by both tooth wear and gingival recessions, dentinal hypersensitivity is also more frequently reported by patients suffering from EDs than controls [44,53].

### 4.3. Oral Mucosal Health

Oral mucosal lesions are often observed in EDs. Factors such as malnutrition and associated deficiencies of vitamins or micro- and macronutrients, dehydration, or pathological behaviors such as provoking vomiting, overbiting, and other parafunctions predispose to their occurrence [59]. Stress levels and addictions like smoking are also important [52].

When analyzing the published results, it is important to pay attention to the profile of the study group. Some differences will exist due to the fact that previous authors analyzed subjects with varying proportions of AN, BN, and EDNOS cases; disease durations; and ages. Panico et al. (2018) [60] and Lesar et al. (2022) [61] observed oral lesions in up to 94% of patients. Their locations can be on the lips, which are dry in as many as 76.2% [62], 93.2% [52], or 69% [28] of subjects. In addition, exfoliative cheilitis, angular cheilitis, and labial erythema are very frequently diagnosed [1,52,53,60,61,63]. The mucosa is anemic, thin, and prone to injury. As a result, it is pale, and more often, ulcerations and hemorrhagic lesions appear on it, the occurrence of which are significantly influenced by the provocation of vomiting and the highly acidic content of vomit [52,60,61,62,63].

Patients are more likely to report mouth irritation, diagnosed as Burning Mouth Syndrome (BMS) [1,53,63]. This disease can be determined by the dentist on the basis of subjective symptoms reported by the patient as it is usually not accompanied by any objective local changes in the oral mucosa. Johansson et al. (2012) [28] showed that the chance of experiencing episodes of burning mouth is 14.2 times higher in patients with eating disorders than in healthy individuals. This is suggested to be associated with mucosal atrophy, xerostomia, and the general disruption of oral homeostasis. Individuals with eating disorders have also been observed to have more common symptoms of cheek and lip biting, as well as impressions on the tongue and linea alba, associated with increased stress levels [52,60,61,63]. A weakened immune system and dysbiosis resulting from a breach in tissue continuity will promote a variety of bacterial, viral, and fungal infections, with the typical clinical presentation of these pathogens [64,65,66,67].

### 4.4. Dental Health

#### 4.4.1. Dental Caries

Eating patterns in EDs that may affect the onset of dental caries include, on the one hand, avoiding entire food groups and certain macronutrients without a medical reason, participating in fad diets to lose weight, and intentionally skipping meals, which may result in nutritional deficiency [68,69]. The low intake of nutrients such as proteins; vitamins A, C, and D; and minerals may enhance their susceptibility to demineralization in carious processes [70]. On the other hand, alternative eating behaviors, such as slowing down the pace of eating, the consumption of large amounts of high-caloric and high-carbohydrate food during binge eating, and engaging in making yourself vomit to control body weight, may contribute to the retention of food debris, the formation of dental plaque on the tooth surface, the lowering of the pH of the oral cavity environment, and the promotion of demineralization, and thus favor dental caries [68]. 

Additional factors that may promote dental caries in ED patients are alterations in the composition of saliva [71]; a decrease in saliva’s buffer capacity and secretion rate [52,53,72,73], which may be associated with the structural changes in the salivary glands [28,74]; self-induced vomiting or starvation; and the side effects of psychotropic drugs (i.e., antidepressants, appetite suppressants), diuretics or laxatives [49]. Undoubtedly, poor oral hygiene in patients with eating disorders, presumably also resulting from psychological reasons, may be a contributing factor to dental caries [47,75]. 

Numerous studies have examined the association between dental caries and EDs, applying commonly used caries assessment measures such as caries prevalence or caries severity based on the clinical or (less often) radiographic recordings and expressed by the sum of decayed (D), missing (M), and filled (F) teeth (DMFT index) or tooth surfaces (DMFS index) [28,47,52,53,74,75,76,77,78] or the International Caries Detection and Assessment System (ICDAS-II) system) [79]. However, the results obtained are contradictory and vary between 37% and 80% depending on the type of ED and the qualification criteria adopted for diagnosis [74,75,78,79,80,81]. Some authors demonstrated significantly higher values in terms of the DMFT index and its D and M components in comparison with control groups [47,53,75]. 

Relationships between the caries index and vomiting frequency are also inconsistent although it is clear that gastric acid along with high-carbohydrate food or beverages may initiate the carious process [82]. 

It is important to emphasize that there is no single ED-associated factor, but rather, all the above-discussed contributing risk factors may have inputs in ED. The following factors should be considered: cariogenic diet, oral hygiene level, remineralization exposure, and taking certain types of medication [81].

#### 4.4.2. Erosive Tooth Wear

**Figure 3:**
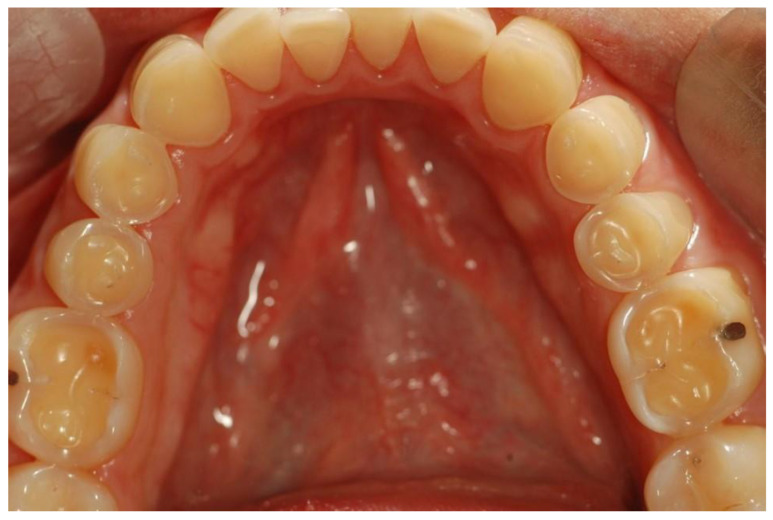
Occlusal surfaces of mandibular teeth affected by erosive teeth wear (Courtsey Amaechi BT, UTHSA, San Antonio, TX, USA).

Erosive tooth wear (dental erosion), the irreversible loss of tooth structure due to chemical dissolution by acids not of bacterial origin, is one of the major oral complications of eating disorders [83]. Erosive tooth wear (ETW) is caused by the frequent contact of teeth with acid from either the stomach (gastric acid) or drinking and eating acidic drinks and foods, particularly outside meal times [84]. EDs, particularly BN, can potentially increase the risk for ETW since they may be associated with bingeing on acidic foods and/or drinks followed by vomiting after every meal [28,29,30,31,85]. Some of these patients use these beverages both in the place of normal meals as well as during intense exercise to lose calories [31]. These characteristics are associated with the frequent contact of the teeth with gastric or dietary acids over an extended period of time, with the consequent wearing away of the dental hard tissue through acid demineralization, initially affecting the enamel, and with the progression to an advanced stage, dentin is exposed (Figure 2 and Figure 3). The exposure of dentin may result in dentin hypersensitivity in response to external stimuli of a cold, hot, tactile, or osmotic nature. The acid of gastric juice brought up due to vomiting, the pH of which can be as low as 1, causes the wear of the palatal surfaces of upper incisors (Figure 2), and with lesion progression, the lingual surfaces of premolars and molars become affected, and in more advanced stages, the process extends to the occlusal surfaces of molars and to the facial surfaces of all teeth [85,86]. Erosion due to dietary acid, with pH ranging from 2.7 to 3.8, has no specific distribution pattern, but depends on factors such as the method of application. Thus, EDs in combination with vomiting are associated with an increased occurrence, severity, and risk of dental erosion [85]. The reported prevalence of ETW among eating disorder patients, particularly those with BN, varies among countries and ranges from 42 to 98% [85].

**Figure 4:**
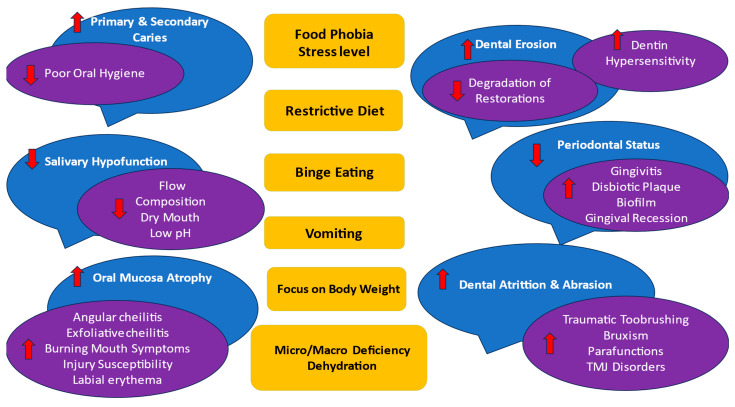
EDs’ main symptoms (in yellow) and oral effects (in blue and purple). The following factors may influence any oral complications: subtype of AN, BN, and EDNOS according to nutritional behaviors; vomiting frequency; disease duration; age and sex of the patient; general health status; pharmacotherapy and their side effects to salivation; and psychosocial profile; as well as cariogenic/acidic diet, individual oral hygiene, and remineralization exposure. Abbreviations: ↑↓ means the ED symptoms increase the oral effect/decrease the oral effect.

Main ED’s symptoms and oral effects are presented in Figure 4.

## 5. Current Approaches to Managing ED

Due to the fact that the effects of EDs on both soft and hard oral tissues are of a multifactorial nature, the dentist may be the first to observe the existence of EDs that may not have been revealed to the medical personnel during history taking. Considering the etiology and symptoms of EDs, management requires a multidisciplinary team involving clinical psychologists, dietitians, and dental professionals. For this reason, the following management should be followed.

### 5.1. Counselling Relating to Dietary Habits

As noted in this manuscript, there exist a wide variety of manifestations of eating disorders. Multiple therapeutic approaches, including psychotherapy, hospitalization, and nutritional counseling, have been employed. A focus on the dietary intake and nutritional education of individuals suffering from eating disorders is of high significance. 

The most severe and aggressive form of an eating disorder is anorexia nervosa. A study of young women who had current disease were assessed as to their state of malnutrition. The study investigated and analyzed their dietary intake with a 4-day food diary recorded by each participant. The nutritional analysis found that their diets were deficient in both macro- and micronutrients. Their body weight and basic mass index (BMI) were lower than in healthy reference groups. The study concluded that all anorexia nervosa patients must undergo nutritional counseling. In severe cases, refeeding may include hospitalization with Nasogastric-tube feedings, nutrition education, and the supplementation of vitamins and minerals to support the refeeding plan [28,29,30].

A meta-analysis of nutritional counseling as part of the clinical approach to treating eating disorders demonstrated the benefit to vary among patients, with some benefitting while others did not, and this was attributed to the varied manifestations of the disease [28,29]. However, a consistent finding indicated that BMI and weight increased with dietetic input. The quality of the diet was positively impacted by nutritional advice. Further, practices and standards conclude that refeeding intervention is necessary and a dietitian/nutritionist is well able to intervene as part of the interprofessional team. Valuable to the outcome was the dietitian’s expertise in selecting a feeding plan to include menus tailored to each patient’s taste and cultural background [28,29].

**Figure 5:**
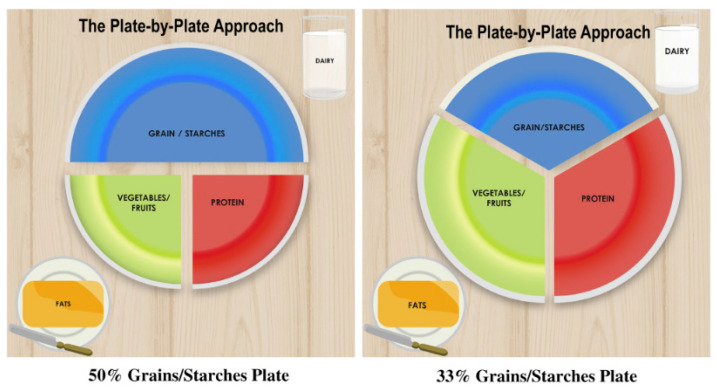
A typical example of the Plate-by-Plate Approach.

In general, the first rule of dietetic therapy is to personalize the refeeding plan of the patient presenting with an eating disorder. Conducting an interview with the patient to determine how they “feel about food” may assist in the education process. Such a conversation may unveil certain aversions to food types, smell, or consistency. The dietitian/nutritionist can prescribe a menu plan that does not include these food items. Furthermore, the omitted food items may be a topic of discussion to dispel any misinformation that may exist [31,87,88]. To initiate the discussion, the dietitian/nutritionist must have the patient’s agreement on the composition of the menu plan. Once agreed upon, the calorie level should be advanced one to two times per week. Weight goals of 2–3 lbs per week are recommended for underweight patients (BMI < 18) [31,88]. It should be noted that eating disorder patients can have normal or above-normal BMI. Nutritional therapy should include support for consistency in the patients’ eating patterns. These menus should include three meals with 1–3 snacks per day. Due to the use of laxatives, purging episodes, and excess exercise, the monitoring of electrolytes, weights, vital signs, and recovery meal plan compliance is advised [31,88]. The recovery meal plan may be a vehicle to give the eating disorder patient “permission” to eat. The goal is to reintroduce food as a partner and not an adversary in the healing process. The MyPlate approach (Figure 5, Table 2) is the simplest place to start with the education process [89,90], and foods to include are:

- Proteins: Total 5 oz/day
- Grains: 6 servings/day
- Dairy or alternative to dairy: 3 servings/day
- Vegetables and fruits: 5 servings/day
- Fats and oils: 4 servings/day

**Table 2:** Seven essential steps and strategies for implementing the Plate-by-Plate Strategy [89,90].

STEP 1	CHOOSE A 10-inch PLATE
STEP 2	PLATE ALL FOOD GROUPS
STEP 3	FILL THE PLATE UP
STEP 4	DECIDE HOW MANY MEALS AND SNACKS
STEP 5	INCLUDE VARIETY
STEP 6	DOES THE MEAL MAKE SENSE?
STEP 7	THE FINAL REVIEW: HOW DOES THE PLATE LOOK? Are all the food groups present? Grains/Starches? Proteins? Fruit/Vegetables? Dairy? Fat? Are the plate 50% grains/starches, 25% protein, 25% fruit/vegetables? (or 33% grains/starches, 33% protein, 33% fruit/vegetable, depending on which plate is recommended?) Is the whole plate full? Have you challenged your child?

The patient must keep an accurate food intake of dairy. The dietitian/nutritionist should review for balance, frequency of meal consumption, variety, and the appropriateness of portion sizes [89,90].

Standard practice for a dietitian’s role in the supervision of a patient with an eating disorder is centered around the core dietetic skills of screening, professional responsibility, assessment, nutrition diagnosis, intervention, monitoring, and evaluation [91]. In summary, regular and careful assessments of nutritional intake along with nutritional counseling can reduce malnutrition in patients with eating disorders. To optimize the chance of full recovery from an eating disorder, a multidisciplinary approach is highly recommended. Currently, dietitians are a part of most eating disorder treatment teams.

### 5.2. Counseling Relating to the Prevention of Effects on Oral Soft Tissues

Faced with this increased risk of plaque-related gingivitis and gingival recession, prevention and early treatment require increased motivation for plaque control and the appropriate teaching of oral hygiene methods. An electric toothbrush is useful in patients with EDs to potentiate plaque removal while controlling brushing time and pressure on the periodontium due to the integrated timer and sensor [89]. 

Regular supportive periodontal care, two times a year, including professional mechanical supra-gingival plaque removal and lifestyle counselling is of the utmost importance for an efficacious preventive approach towards periodontal complications in ED patients.

### 5.3. Counseling Relating to the Prevention of Effects on Oral Hard Tissues (Dental Caries)

Individuals suffering from EDs are included in the high-caries-risk group [92]. Therefore, effective strategies for preventing dental caries should be applied (Table 3) [93].

While fluoride compounds, applied to remineralize dental hard tissues, need the salivary calcium and phosphate ions, in the case of ED hyposalivation, they may diminish the remineralization process. Apart from fluoride compounds, the cariostatic effect of fluoride-free formulations such as calcium phosphates, either in amorphous or crystalline form (hydroxyapatite), has also been used with advantageous results in several long-term randomized clinical trials [94,95,96,97,98,99]. The mode of action of hydroxyapatite involves providing mineral ions required for remineralization. These deposits form a protective layer on the enamel surface, eliminate the harmful effects of cariogenic acids, and prevent the adhesion of plaque-forming bacteria to the tooth surface [100]. 

**Table 3:** The main strategies for preventing dental caries.

Control the bacterial biofilm	via physical methods (toothbrush, interdental and tongue brushes, dental floss, irrigators) along with antibacterial preparations if temporarily needed (toothpaste or mouthwashes containing active agents, e.g., chlorhexidine, stannous salts, or zinc salts)
Increase in the resistance of hard dental tissues to the demineralizing effect of bacterial acids	via the topical use of remineralizing agents (at home and in-office application)
Modification of the diet	limiting the consumption of fermentable carbohydrates; avoiding sticky/acid products and replacing them with caries-protective foods such as raw vegetables, nuts, and cheese
Stimulation of salivary flow	with consistent food and sugar-free chewing gums containing sucrose substitutes i.e., xylitol
Altering medication-induced hyposalivation if needed [101,102,103]	
Regular dental check-ups every 3 months to monitor the oral condition and motivate the patient	

### 5.4. Counseling Relating to the Prevention of Effects on Oral Hard Tissues (Erosive Tooth Wear)

Once dental erosion is detected, there is a need for a full case history, which should include dietary history, medical history, dental hygiene habits, and lifestyle history. This would establish the risk factors and help in the development of individualized counseling. Erosive tooth wear management in ED patients must involve education (counselling) on risk factors and the prevention of occurrence and protection of affected surfaces from further damage, and in cases of severe structural damage, restoration may be required.

The counseling of ED patients should start by educating each patient on the risk factors predisposing him/her to ETW, with a referral for psychoeducation and dietary advice. Eating disorder patients should take the following precautions [83].

When possible, ‘bite-guards’ should be worn while vomiting. The inside (tooth surface) of the guard should be coated with a small amount of Sodium Bicarbonate suspension, or Milk of Magnesia, to neutralize any gastric acid pooling in it.

Patients must avoid toothbrushing immediately after each episode of vomiting or bingeing on acidic food or drink; rather, patients should use any of the following to freshen their mouth and wait for at least 60 min before toothbrushing [104]: fluoride mouthwash, fluoride tablets/lozenges or dairy products (e.g., milk) to enhance the rapid remineralization of the softened tooth surface, sugar-free chewing gum or lozenges to increase saliva flow to neutralize the acidity and provide an alkaline environment to facilitate the rapid remineralization of the softened tooth tissue, and sugar-free antacid tablets or a pinch of sodium bicarbonate (or baking soda) dissolved in some water to neutralize the acidic oral fluid.

They must use toothpaste containing either high fluoride concentrations or stabilized stannous fluoride, as well as fluoride mouth rinses, for their routine daily oral hygiene practice.

The intake of acidic dietary products with added calcium reduces the erosive effect of the drink [105].

### 5.5. Steps to Protect Affected Oral Soft and Hard Tissues

Current recommendations for oral management of patients with EDs are based on the need for early intervention without waiting for the remission/curing of eating symptoms, which sometimes takes years [106]. Oral treatment is part of the comprehensive, multidisciplinary, and personalized care project that is coordinated by the general practitioner and the psychiatrist. After making a differential diagnosis with the usual dental pathologies (carious lesions, fractures, cracks, defective restorative care), the positive diagnosis of dentinal hypersensitivity associated with gingival recessions will be confirmed via the visual inspection of dentinal exposure sites, most often at the cervical level that is sensitive to contact (tactile probe test) and/or air using the Schiff test, which is graded from 0 to 3 [107]. Non-invasive treatment is preferred and may include a prescription of desensitizing agents in the form of toothpaste and/or mouthwash. However, in many situations, in patients with an ED, the advanced stage of the hard and soft tissue lesions will indicate restorative treatment and/or periodontal plastic surgery as part of comprehensive management [108]. However, no study has explored the safety and efficacy of root coverage procedures in ED patients. Tobacco cessation has a beneficial impact on root coverage success and ED patients who smoke have to be encouraged to quit before periodontal plastic surgeries [109].

Eroded tooth surfaces in ED patients can be protected from further erosive damage and deterioration in appearance by using any of the following procedures. Dentin bonding agents can be applied to protect erosively exposed dentinal tissues to reduce the rate of tooth wear [110]. Adhesively retained resin veneers and crowns both improve appearance as well as provide protection against further damage [111,112]. These restorations are easy to place in advanced cases with dentinal exposure when the eating symptoms are not fully controlled [113,114]. Porcelain restorations may be preferred when the patient recovers from the ED.

### 5.6. Recall Visits for Continued Care to Maintain Compliance and Oral Health

**Figure 6:**
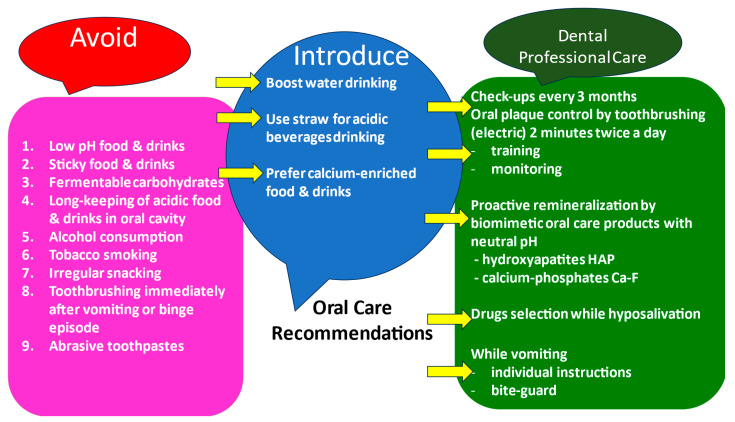
Counseling relating to the prevention of effects on oral hard and soft tissues (what to avoid but introduce for the patient and professional care).

A continued care regime matched to the patient’s requirements should be established to check patient compliance, monitor wear, reinforce advice, and provide encouragement to maintain changed behavior. Failure to monitor the patient over time may result in a relapse of the condition (Figure 6).

## 6. Conclusions

Based on the specific psychopathological ED symptoms associated with food intake, it is possible to identify EDs that lead to a number of somatic complications and changes in the functioning of the oral cavity. The present review covers various directions in which dietary habits and oral health may be altered during the course of an ED. However, oral complications in ED patients are of multifactorial origin, and as such, their management requires multidisciplinary approaches. Current concepts in oral disease prevention should focus on tooth remineralization and the control of all the key factors involved in oral complications. The results of this review highlight the role that should be played in this regard by groups of nutritionists, dental professionals, and medical practitioners. Therefore, it should be considered as a serious and complex health problem. The present study uncovers analysis that future investigations may be forwarded. Further studies, preferably multi-center longitudinal studies involving larger populations with EDs, are necessary to establish, precisely, the association between dietary patterns in ED patients and their oral factors, e.g., microbiota. To inhibit oral repercussions, we recommend tailored interventions consisting of oral plaque control, moisturizing oral mucosa, intensive remineralization based not only on fluoride but also hydroxyapatites and calcium-phosphate products, and preventive advice and bite-guard application while vomiting. 

## Figures and Tables

**Table 1:** List of binge foods reported by patients: composition and NOVA categories [31].

	Carbohydrate %	Fat%	Protein%	NOVA Category
**Ice cream**	47	46	7	4
**Doughnut**	40–45	45–50	2–3	4
**Pita bread and hummus**	50	39	11	4
**Raisin bagels**	80	6	8	4
**Cookies**	60	38	2	4
**Nuts**	12	75	13	1 or 4
**Diet soda**	Artificial sweetener	0	0	4
**Potato chips**	35	60	5	4
**Chocolate cake**	57	40	3	4
**Cherry yoghurt**	75	10	15	4
**Pizza**	48	37	15	4

NOVA: a classification of food products that assigns the level of processing by the food industry. Examples are described as categories such as minimally processed and ultra-processed. Numerical categories defined: Group 1 comprises foods that are unprocessed or minimally processed. Examples: salt, sugar, oil, and fats. Group 2 is defined as comprising foods from Group 1 with minimal processing with culinary ingredients. Examples are oils, butter, lard, sugar, and salt. Group 3 comprises processed foods, i.e., products manufactured by industry with added substances to increase palatability or shelf stability. Examples include bottled vegetables or legumes preserved in brine and vinegar, fruits in syrup, meat products and canned fish, smoked fish, freshly baked bread, and simple cheeses to which salt is added. Group 4: Ultra-processed foods formulated with ingredients exclusive to industry. Examples are soft drinks, sweets, salty or fatty foods, packaged snacks, candy, pasties, breakfast cereals, and energy drinks.

## Data Availability

Data associated with the paper are not publicly available but are available from the corresponding author on reasonable request.
